# Quality and Nutritional/Textural Properties of Durum Wheat Pasta Enriched with Cricket Powder

**DOI:** 10.3390/foods8020046

**Published:** 2019-02-01

**Authors:** Adamina Duda, Julia Adamczak, Paulina Chełmińska, Justyna Juszkiewicz, Przemysław Kowalczewski

**Affiliations:** 1 Students’ Scientific Club of Food Technologists, Faculty of Food Science and Nutrition, Poznań University of Life Sciences, 60-637 Poznań, Poland; adaminaduda@wp.pl (A.D.); juliaadamczak1@gmail.com (J.A.); paulina.chelminska1@gmail.com (P.C.); justyna.juszkiewicz@wp.pl (J.J.); 2 Institute of Food Technology of Plant Origin, Faculty of Food Science and Nutrition, Poznań University of Life Sciences, 60-637 Poznań, Poland

**Keywords:** novel food, insects, high-protein pasta, protein enrichment, texture, consumer acceptance

## Abstract

Cricket powder (CP) contains significant amounts of protein, fat (including unsaturated fatty acids), and fiber, as well as vitamins and minerals. The high nutritional value and low price make it an interesting addition to food production. This paper is a report on the results of the addition of cricket powder to pasta. Three levels of durum semolina replacement were chosen: 5%, 10%, and 15%. The obtained products were analyzed for their nutritional composition, cooking and textural properties, and color, as well as consumer acceptance. The results indicate that the addition of CP influenced the cooking weight and cooking loss (reducing losses and water absorption), as well as the color of the pasta, reducing its lightness and shifting color balances to blue and red. The firmness of pasta was also influenced. The firmness was strengthened by addition of CP. Principal components analysis indicated that the flavor change had the most pronounced effect on consumer acceptance. Nevertheless, sensory evaluation proved that protein-enriched pasta produced with CP has consumer acceptance comparable with that of conventional products.

## 1. Introduction

Pasta is cheap to produce and very convenient to prepare, which makes it one of the most popular high-carbohydrate food products [1]. Due to the multitude of ways of preparation and universal character, pasta can be eaten without causing consumer reluctance to eat it constantly. Pasta contains carbohydrates (from 74% to 77%) and protein (from 11% to 15%). Nevertheless, the quality of pasta protein is low because of the limitations in the amounts of essential amino acids, notably lysine [2]. Traditionally, wheat semolina has been the primary ingredient of pasta. However, components that increase nutritional value or exert a beneficial effect on health can be used in its production [3,4]. The use of additives that contain limiting amino acids increases the nutritional value of the pasta significantly. Cricket powder is an interesting additive.

Edible insects are commonly used as food in Africa, Latin America, and Asia [5]. Because of their significant content of protein, vitamins, and minerals [6] the Food and Agriculture Organization of the United Nations (FAO) recommends the consumption of edible insects [7]. Crickets, mealy larvae, ants, grasshoppers, and flies are some examples [6]. In Europe, however, there is no tradition of using insects as food [5]. Until recently, some European member states (e.g., Italy, Spain, Ireland) considered whole insects and their parts novel foods. Other member states (the United Kingdom, Denmark, the Netherlands, Belgium) did not have regulations regarding edible insects and only considered substances isolated from insects (such as proteins, lipids) as novel foods. From 1 January 2018, insects as well as their parts can officially be regarded as so-called “novel foods”. The change in legislation in this area may contribute to the increase of insect consumption in Europe [8,9]. Of note, the European Food Safety Authority published an analysis of the risks associated with the use of insects as food products, pointing to potential microbiological, chemical, or environmental risks, recommending the monitoring of edible insects, especially those coming from outside the European Union [10].

Due to high nutritional value, crickets and cricket powder obtained from them may be considered a valuable protein additive in the production of food. They contain significant amounts of proteins, lipids (especially polyunsaturated fatty acids) and minerals, such as calcium, iron, and zinc [11,12]. However, depending on the type of insect, its development stage, and type of breeding (insect farm or caught in the wild), the protein content varies considerably, from 13% to over 77% of dry matter [13]. Country of origin, environmental conditions, and type of heat treatment (boiling, baking, roasting) also deeply affect the nutritional value of edible insects [14]. Stull et al. [15] state that cricket powder may stimulate the growth of intestinal microbiota and decrease the level of tumor necrosis factor alpha (TNF-α) in plasma. Additionally, chitin and chitosan, substances abundant in crickets, were found to suppress pathogenic microorganisms in the intestines [16]. The published results also indicate high antioxidant and anti-inflammatory activity of insect proteins subjected to enzymatic hydrolysis [17,18].

Compared to other proteins of animal origin (poultry, pork, or beef), the production of insect protein has a smaller environmental footprint and a higher economic value. In order to produce protein from insects, much less water is needed. Moreover, much smaller areas of land are necessary for its production [6]. The possibility of using waste products from the agri-food industry as feed is another advantage. It facilitates a reduction of production-associated costs and the process is independent of the feedstock used as food by humans [19]. Additionally, such production also generates smaller quantities of gases harmful to the environment, such as CO_2_ or methane [20]. All these benefits make cricket-based additives not only an additive that is inexpensive, healthy, and functional, but also environmentally-friendly.

Unfortunately, there is a reluctance on behalf a part of consumers regarding the use of insects as food. Because of this, processing crickets into cricket powder is necessary in order to gain acceptability. It also needs to be stressed that the use of additives for the production of pasta, especially this rich in protein, may result in changes not only in the culinary traits of pasta, but also in the texture [21,22]. The firmness of pasta and its low stickiness are important qualities for consumers [23]. Additives such as whey [24] or protein of legumes [1,21,25], may improve the nutritional value on the one hand, but on the other, they may change the consistency of the pasta dough. This results in difficulties in obtaining an enriched product of proper quality that would be acceptable to consumers. 

Therefore, the aim of this work was to evaluate the influence of the addition of cricket powder on the nutritional value, texture, and characteristics of pasta. Consumer acceptance of pasta was examined as well.

## 2. Materials and Methods 

### 2.1. Pasta Manufacturing

Spaghetti was prepared with the use of a pasta extruder Häussler Emma (Karl-Heinz Häussler GmbH, Heiligkreuztal, Germany). Semolina of *Triticum durum* was obtained from a commercial mill (Radzyń Podlaski, Poland), while the cricket powder (CP) was bought from Eat Grub Ltd. (London, UK). Each of the three formulations consisted of semolina and cricket powder in different proportions were hydrated to obtain 33% content of water, mixed for 30 min and then extruded. Fresh pasta was dried in a humidity chamber (TH-TG-180, Jeio Tech, Seoul, Korea). The drying parameters were as follows: 45 °C, Φ = 75% for 3 h; 63 °C Φ = 85% for 12 h; 40 °C Φ = 60% for 4 h. Dried pasta was left for 10 h at room temperature and 70% relative humidity to stabilize, and after that pasta was packed into polyethylene (PE) bags. The final pasta formulations contained 5%, 10%, or 15% addition of CP, and were named as CP5, CP10, and CP15, respectively. Pasta without CP addition was used as a reference (R). Before the analyses, pasta was cooked in a tap water (water/pasta ratio of 10, 6 min). 

### 2.2. Nutritional Composition and Energy Value

Total nitrogen content was determined by the Kjeldahl method according to ISO 20483 [26] and was used to calculate the protein content by multiplying the result by the conversion factor of 5.7. The fat content (Soxhlet method) was determined according to American Association for Clinical Chemistry (AACC) 30-25.01 [27], ash content according to AACC method 08-12.01 [28] and moisture content according to AACC 44-19.01 [29]. Moreover, the proximate carbohydrate content was estimated by subtracting the total fat, protein, ash and moisture content from 100%. The energy value (EV) was calculated with the following formula:

EV (kcal/100 g) = 4 × protein (%) + 4 × carbohydrate (%) + 9 × fat (%)(1)

### 2.3. Color Measurements

Color of cooked pasta was measured using the Chroma Meter CR-410 (Konica Minolta Sensing Inc., Osaka, Japan) color meter. Differences were recorded in CIE L*a*b* scale in terms of lightness (L*) and color (a*—redness; b*—yellowness) [30]. Moreover, total color difference (ΔE) was calculated using Formula 2 [31].

(2) $$\Delta E=\sqrt{\Delta L^{*2}+\Delta a^{*2}+\Delta b^{*2}}$$

### 2.4. Optimal Cooking Time

The optimal cooking time for the pasta samples was determined in accordance with the method described by Phongthai et al. [32]. Dry pasta samples were boiled in water (ratio 1:10) and the optimal cooking time was monitored during cooking until the white core of the pasta samples (visible in cross-section) disappeared.

### 2.5. Cooking Behaviors

Cooking weight (CW) and cooking loss (CL) were determined according to Zardetto et al. [33]. Previously weighed pasta (about 50 g) was cooked in 500 mL of water. After that, the pasta sample was removed, drained, and weighed after 3 min. CW was calculated as mass ratio of cooked and raw pasta (g/g). The drain water was collected in a tarred beaker, placed in an air oven at 105 °C and evaporated to dryness. The residue was weighed and reported as a percentage of dry pasta (CL).

### 2.6. Texture Analysis

Texture analysis was analyzed using a TA.XTplus texture analyzer (Stable Micro System Co. Ltd., Godalming, UK) equipped with a 5-kg load cell and the Pasta Quality Ring made of plexiglass (A/LKB-F, Stable Micro System Co. Ltd., Godalming, UK). Five pieces of cooked pasta were placed centrally under the knife blade onto the measurement plate of the texture analyzer (*n* = 15). The measurement was performed at the test speed: 0.17 mm/s and the posttest speed: 10 mm/s. The firmness of pasta was recorded as the maximum force required to cut the sample. In addition, the work needed to move the blade thought the sample was calculated [34].

### 2.7. Consumer Acceptance

The rating of consumer acceptance was measured using the 9-point hedonic line scale (ranging from 1 as being “dislike very much” to 9 as being “like very much”) [35]. In this study, 20 untrained panelists, aged between 20 and 40 years, were invited to participate. Consumers were asked to evaluate the appearance, color, flavor, taste, texture, and overall rating of analyzed pasta.

### 2.8. Statistical Analysis

All the measurements were repeated three times, unless stated otherwise. One-way analysis of variance (ANOVA) was carried out independently for each dependent variable. A post-hoc Tukey’s HSD multiple comparison test was used to identify statistically homogeneous subsets α = 0.05. Principal component analysis (PCA) was performed using all the data obtained in the analyses. The result is presented in a two-dimensional system (biplot) obtained by plotting the observations and variables on the plane formed by the calculated principal components. Additionally, a correlation matrix was constructed for the variables used in PCA. Statistical analysis as well as PCA were performed with Statistica 13 software (Dell Software Inc., Round Rock, TX, USA).

## 3. Results and Discussion

### 3.1. Nutritional Value of Pasta

The nutritional value and energy content of pasta depend on the type of raw materials used for its production. To improve the value of pasta, additives, such as protein concentrates, yeast, milk and vegetable additives, are used [4,21,32]. Cricket powder is a high-protein product that also contains significant amounts of fat and minerals [6,11]. It was shown that the addition of CP caused a significant increase in the content of protein in pasta from 9.96% for R, to 16.92% for CP15 (Table 1). As could have been expected, the content of fat and minerals increased with increasing amounts of the CP additive. Fat content increased from 1.31% (R) to 4.73% (CP15), and minerals content from 0.86% (R) of to 1.46% (CP15). However, the carbohydrate content decreased. Lower carbohydrate content accompanied by higher fat and protein contents makes the enriched pasta an interesting product for athletes [36,37].

### 3.2. Color of Pasta

Consumers assessment of food is based not only on the nutritional value or potential pro-health effects but also on sensory features which directly affect consumer preferences, selection and desires [38,39,40]. Among these sensory properties, color is one of the most easily evaluated characteristics. The use of CP additive for the production of pasta resulted in distinct differences in color, which persisted also after the cooking process. The pasta samples that contained CP were clearly darker (Table 2). Their color was similar to commercially available whole wheat pasta, widely considered to be healthier. The results of the color analysis showed that the value of L* parameter, related directly with the lightness of the pasta, decreased by almost 50% in the case of the CP15 sample. Moreover, color balance was shifted towards red (positive a*) and blue (negative b*) in all the enriched pasta samples. The more CP was used, the more pronounced the changes were. Total color differences (ΔE), which represent the magnitude of the color difference between the reference pasta and pasta with CP, were in the range from 27.18 to 41.29 (*p* < 0.05). Bellary et al. [41] pointed out that a color difference is perceivable to the naked eye when ΔE > 3.0. Other data also indicates that an inexperienced observer can visually detect color deviation when ΔE is greater than 3.5 between two objects [31]. In the case of all the analyzed pasta samples, ΔE was found to be higher than 25. This indicates that the alteration of color that results from the use of the additive is strong enough to be visible. Such a significant difference in color can result in a reduction of the attractiveness to consumers [38].

### 3.3. Cooking Behaviors

Cooking properties are important indicators of pasta quality. A change in the recipe of pasta, in particular the addition of protein or fiber, can significantly affect its properties [4,42]. Cooking properties of pasta enriched with CP are presented in Table 3. The addition of CP caused an increase of the optimal cooking time from 6 min (R) to 7 min (CP15), simultaneously lowering both cooking weight and cooking loss. As indicated by Larrosa et al. [43] the lower the cooking loss, the higher the quality of the pasta. Generally, the use of protein additives of plant origin was found to increase cooking losses [44]. On the contrary, the addition of CP resulted in reduced losses. This could be interpreted as an indicator of better quality of the final product.

### 3.4. Textural Properties

The texture of pasta is the most important indicator of quality that influences consumer acceptance [23]. Analysis of the texture of cooked pasta was carried out by examining two parameters: firmness and total work required to completely cut the sample. The firmness of pasta depends not only on the conditions of its production, but also on the time of cooking [45]. To compare the textural properties of the pasta with CP, optimal cooking time was determined for each sample. This allowed the analyzes to be carried out in a manner that was free from the influence of cooking time. The firmness as well as the total work of shear are presented in Table 4. The results of texture analysis indicate that the firmness of pasta samples enriched in CP was higher than that of the control sample. Data in the literature indicate that protein additives used in the production of pasta, such as egg and broad bean protein, increase the firmness, which is consistent with the obtained results [46]. Moreover, Kim and Wiesenborn [47] stated that the firmness of pasta was significantly correlated to cooking loss. Increased firmness seemed to limit the swelling of starch granules, partly contributing to the reduction of cooking loss (Table 3). Moreover, the combination of high firmness and small losses during cooking are characteristic of high quality pasta [48].

### 3.5. Consumer Study

**Figure 1:**
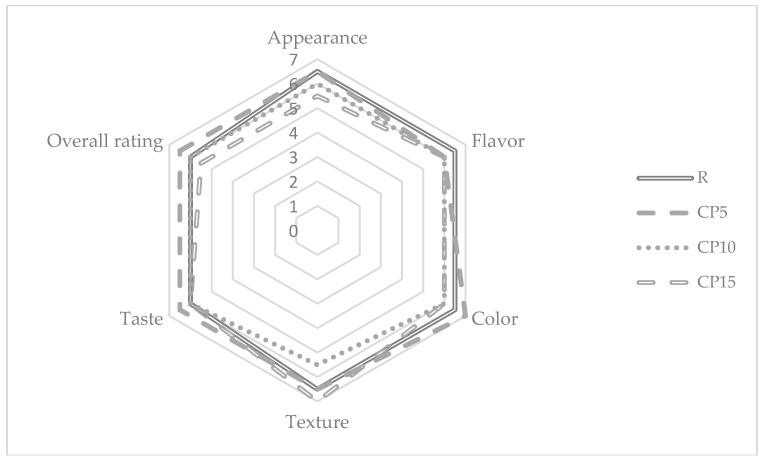
Consumer acceptance of obtained pasta. R, CP5, CP10, and CP15 denote pasta with cricket powder at 0%, 5%, 10%, and 15% (*w*/*w*), respectively.

Visual properties, such as color or appearance, often influence the decision to buy a product by consumers [3]. Based on the results of the sensory analysis, it was shown that the CP additive significantly changed the scores given by consumers (Figure 1). The pasta with the highest level of the additive significantly differed in the assessment from the other variants. Interestingly, the texture in this case was rated better than even the reference sample. Nevertheless, a clear foreign flavor and dark color contributed to the lower scores. A small addition of CP (5%) made, however, that the color of the pasta was evaluated better. This pasta looked like a wholemeal pasta, commonly associated with a healthier one. The texture and taste of this variant also received high scores. The use of a small amount of the additive allows for obtaining pasta with high consumer acceptance.

### 3.6. Principal Component Analysis

**Figure 2:**
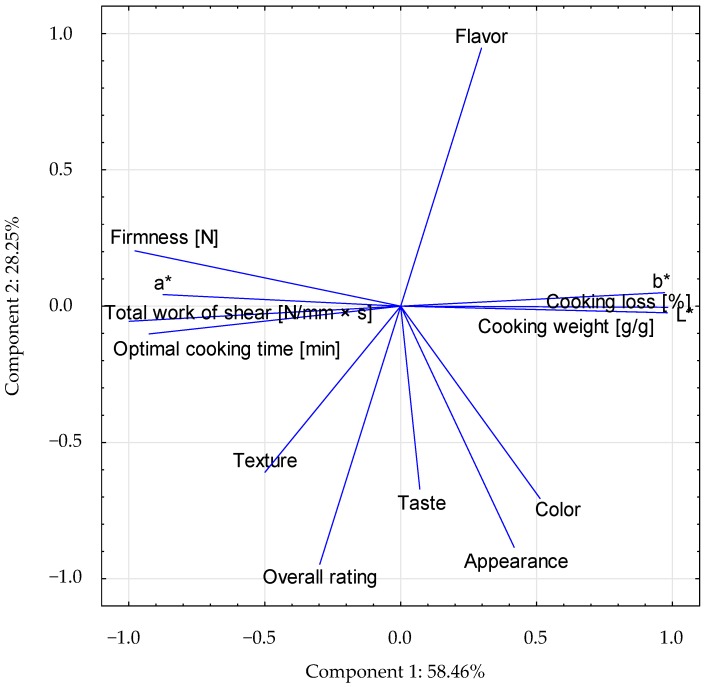
Projection of the variables on the component plane (1 × 2). L*: lightness; a*: redness; b*: yellowness.

To find the relationships between the analyzed physicochemical and sensory parameters, a Principal component analysis (PCA) analysis was performed. PCA analysis allows for the determination of variables that exert the greatest impact on the appearance of individual components. The presented PCA analysis facilitates the interpretation of the impact of CP additive and physicochemical properties of pasta on consumer acceptance. Two main components derived from the calculations were selected; they allowed for the explanation of 86.71% of the total variance of 13 analyzed variables. The first main component contains 58.46% of the information about the tested products represented by variables, while the second main component contains 28.25% of the information. Relations between variables are shown in Figure 2. Each of the vectors represents one variable, and its size and direction, describes the effect it exerts on the main components. The first component describes the sensory characteristics (taste, appearance, color, smell, texture, overall rating). The odor-related vector has a direction opposite to the rest of the parameters assessed in sensory analysis. This means that there was a negative correlation between these variables and the flavor rating of pasta with the amount of CP added. The second component describes the instrumental structure analysis (firmness, total work of shear), the color of the pasta (L*, a*, b*) and cooking parameters (cooking loss, cooking weight, and optimal cooking time as well). The model describing the color of pasta shows relatively long vectors, which indicates that individual samples were significantly distinct in relation to each other. This is consistent with the fact that the color differences were visible to the naked eye. The distinguishing vector is flavor, which plays an important role in distinguishing the pasta samples. Details concerning correlations between the variables are included in Table S1.

## 4. Conclusions

The increase in population and per capita consumption has resulted in a growing demand for available resources in order to meet the nutritional needs of both animals and humans. In the face of the increasing needs, insects are looked upon as a raw material for the production of food with high nutritional value because of their significant content of protein, fat and minerals. Insects have been consumed in Asian, African, and Latin American countries for a long time. Recent trends indicate that edible insects could also gain increased attention in Europe. As consumption of whole crickets is unacceptable to many, the use of powder obtained from crickets is justified. The impact of cricket powder additive on the nutritional value and quality of pasta was investigated in this paper. It was shown that the additive changed the characteristics of the enriched pasta significantly. It was found that the addition of CP in the lowest studied amount of 5% caused a significant increase in the content of protein, fat, and mineral content in comparison to pasta obtained without CP. At the same time, it was shown that the enriched pasta had different cooking properties. The addition of CP resulted in a decrease in cooking weight and cooking loss, but caused an increase of the optimal cooking time. In the case of color analysis, the lightness of the pasta decreased (value of the L* parameter), and the color balance was changed as well (shifted towards red and blue) with the introduction of CP. Enriched pastas were also characterized by higher firmness than the reference pasta, which, in combination with lower cooking loss values, is proof of the high quality of the product. Consumer evaluation showed that the use of the CP additive was met with high acceptance. The color of pasta sample with a 5% CP addition was described as resembling wholemeal pasta by the consumers. Such appearance is associated with an impression of a healthy product. Higher scores were also given for taste and overall rating. Application of CP at the amount of 5% allows for obtaining pasta with increased content of protein and mineral compounds, improved culinary properties, and texture that is additionally highly attractive to consumers.

## Figures and Tables

**Table 1:** Nutritional value of pasta with cricket powder (CP).

Parameter	R	CP5	CP10	CP15
Protein (%)	9.96 ± 0.92 ^d^	12.27 ± 0.88 ^c^	14.60 ± 0.93 ^b^	16.92 ± 1.01 ^a^
Fat (%)	1.31 ± 0.03 ^d^	2.45 ± 0.10 ^c^	3.59 ± 0.21 ^b^	4.73 ± 0.32 ^a^
Ash (%)	0.86 ± 0.03 ^c^	1.04 ± 0.01 ^b^	1.25 ± 0.09 ^a^	1.46 ± 0.08 ^a^
Carbohydrate ^1^ (%)	84.73 ± 1.63 ^a^	81.23 ± 1.81 ^b^	76.57 ± 2.11 ^c^	73.42 ± 1.02 ^d^
Energy value ^2^ (kcal/100 g)	390.55 ± 2.33 ^c^	396.09 ± 1.92 ^b^	396.99 ± 2.11 ^ab^	403.93 ± 2.68 ^a^

R, CP5, CP10, and CP15 denote pasta with cricket powder at 0%, 5%, 10%, and 15% (*w*/*w*), respectively. ^1^ The carbohydrate content was estimated by subtracting the average content of ash, fat, and protein from 100%. ^2^ Energy value was calculated based on average protein, fat and carbohydrate content. Mean values denoted by different letters (a–d) differ statistically significantly (*p* < 0.05).

**Table 2:** Color parameters of analyzed pasta samples.

Parameter	R	CP5	CP10	CP15
L*	78.82 ± 0.14 ^a^	55.39 ± 0.10 ^b^	51.22 ± 0.11 ^c^	41.34 ± 0.90 ^d^
a*	−3.67 ± 0.03 ^d^	6.77 ± 0.02 ^c^	7.42 ± 0.01 ^b^	7.74 ± 0.04 ^a^
b*	22.04 ± 0.03 ^a^	13.06 ± 0.09 ^b^	12.72 ± 0.01 ^c^	9.00 ± 0.03 ^d^
ΔE	-	27.18	31.17	41.29

R, CP5, CP10, and CP15 denote pasta with cricket powder at 0%, 5%, 10%, and 15% (*w*/*w*), respectively. Mean values denoted by different letters (a–d) differ statistically significantly (*p* < 0.05). L*: lightness; a*: redness; b*: yellowness; ΔE: total color difference.

**Table 3:** Cooking properties of durum wheat pasta enriched with cricket powder.

Parameter	R	CP5	CP10	CP15
Optimal cooking time (min)	6.05 ± 0.05 ^c^	6.25 ± 0.11 ^b^	6.35 ± 0.09 ^b^	7.03 ± 0.08 ^a^
Cooking weight (g/g)	2.74 ± 0.12 ^a^	1.86 ± 0.09 ^b^	1.78 ± 0.10 ^c^	1.61 ± 0.17 ^c^
Cooking loss (%)	3.94 ± 0.01 ^a^	3.30 ± 0.01 ^b^	2.96 ± 0.08 ^c^	1.96 ± 0.06 ^d^

R, CP5, CP10, and CP15 denote pasta with cricket powder at 0%, 5%, 10%, and 15% (*w*/*w*), respectively. Mean values denoted by different letters (a–d) differ statistically significantly (*p* < 0.05).

**Table 4:** Texture of pasta.

Parameter.	R	CP5	CP10	CP15
Firmness (N)	3.25 ± 0.27 ^b^	3.46 ± 0.92 ^b^	4.13 ± 0.94 ^ab^	4.97 ± 0.81 ^a^
Total work of shear (N/mm × s)	0.32 ± 0.01 ^a^	0.33 ± 0.02 ^a^	0.33 ± 0.02 ^a^	0.33 ± 0.02 ^a^

R, CP5, CP10, and CP15 denote pasta with cricket powder at 0%, 5%, 10%, and 15% (*w*/*w*), respectively. Mean values denoted by different letters (a, b) differ statistically significantly (*p* < 0.05).

## Supplementary Materials

The following are available online at http://www.mdpi.com/2304-8158/8/2/46/s1, Table S1: Correlations matrix for investigated variables (*p* < 0.05).
